# Lycopene Increases Skeletal Muscle Mass by Promoting Myoblast Differentiation

**DOI:** 10.1002/fsn3.72252

**Published:** 2026-08-10

**Authors:** Wanxue Wen, Dajiang Ding, Junning Pu, Yong Zhang, Yexia Luo, Jiaxin Du, Daolin Mou

**Affiliations:** ^1^ School of Food and Liquor Engineering (School of Wuliangye Baijiu) Sichuan University of Science and Engineering Yibin Sichuan China; ^2^ Institute of Neuroscience Chinese Academy of Sciences Shanghai China; ^3^ Institute of Animal Nutrition Sichuan Agricultural University Chengdu Sichuan China; ^4^ Southwest University of Science and Technology Mianyang Sichuan China

**Keywords:** C2C12, ERα, lycopene, muscle mass, myoblast differentiation

## Abstract

Muscle differentiation is a necessary process for the growth, development and regeneration of skeletal muscles. Lycopene has been extensively documented to play a critical role in promoting mitochondrial biogenesis and energy metabolism in skeletal muscle. However, the role of lycopene, as a nutrient‐active compound, in the process of muscle differentiation has not yet been reported. Therefore, this study aimed to assess the effects of supplementation with lycopene on skeletal muscle differentiation in vivo and in vitro, and investigate its potential mechanism. Results demonstrated that lycopene‐fed mice showed markedly increased gastrocnemius muscle (GAS) mass and myofiber size. Lycopene promoted muscle protein synthesis and upregulated the expression levels of myogenic regulatory factors such as MyoD (myogenic determining factor) and MyoG (myogenin) in mice and in C2C12 cells, accompanied by promoted myotube formation during cell differentiation. Transcriptome sequencing analysis revealed that lycopene‐induced promotion of muscle differentiation was associated with enhanced activity in the estrogen signaling pathway, as evidenced by upregulated expression levels of ERα in both mice and myoblasts. Furthermore, pharmacological inhibition or ERα specific‐knockdown was used to analyze its function in muscle differentiation. Results found that ERα suppression effectively hindered the formation of myotubes and abolished the pro‐differentiation effects of lycopene. Taken together, these findings suggested that lycopene exhibited beneficial effects on myoblast differentiation contributing to increased muscle mass.

## Graphical abstract

Lycopene supplementation could increase skeletal muscle mass by promoting myoblast differentiation and muscle protein synthesis. Mechanistic analysis revealed that lycopene could activate ERα, and ERα activation facilitated myogenic differentiation and myotube formation.

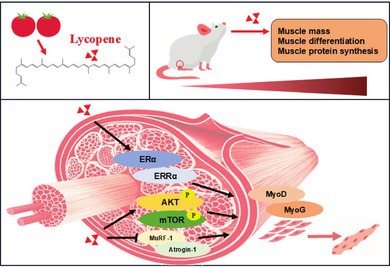

## 1 Introduction

Skeletal muscle is essential for physiological activities and metabolism and it occupies approximately 40%–50% of the whole body mass (Li et al. 2024). Loss of skeletal muscle leads to muscle atrophy and metabolic diseases (Cohen et al. 2015; Sartori et al. 2021). Thus, skeletal muscle maintenance is essential for keeping overall body health and determining the quality of life (Sayer et al. 2024). Skeletal muscle is composed of large multinucleated cells, and muscle formation and regeneration involve several steps including myoblast proliferation, differentiation, and fusion of myogenic cells into multinuclear myotubes and the formation of muscle fibers (Buckingham 2006; Dumont et al. 2015; Tieland et al. 2018). Among them, myoblast differentiation represents a necessary and critical step in myogenesis. The process is regulated by MRFs (myogenic regulatory factors) including MyoD, Myf5, MyoG, and MRF4 (Dumont et al. 2015; Rudnicki and Jaenisch 1995; Zammit 2017). MyoD specifies myogenic lineage commitment during early myogenesis, whereas MyoG drives terminal differentiation of myoblasts into mature myotubes (Arnold and Winter 1998; Jiang et al. 2023). Both transcription factors cooperatively upregulate the expression of myosin heavy chain (MHC, muscle‐specific gene) and enhance CK (creatine kinase) enzyme activity, thereby orchestrating the coordinated progression of skeletal muscle differentiation (Sabourin and Rudnicki 2000). Researches have also reported that both nutrition and genetic regulators critically govern skeletal muscle differentiation (Beaudry et al. 2016; Florini et al. 1991; Tieland et al. 2018). For example, angiopoietin‐like protein‐4 could inhibit muscle differentiation through decreasing the Wnt/β‐catenin pathway (Son et al. 2023). Treated with glucosamine inhibited C2C12 differentiation and promoted myotube atrophy through distinct signal pathways (Liu et al. 2025).

Lycopene is one of the strongest antioxidants in nature and can be extracted from guavas, tomatoes or watermelons. It has been reported that supplementation with lycopene confers multiple physiological benefits, including potent antioxidant activity and enhanced anti‐inflammatory capacity (Mou et al. 2025; Wen et al. 2021). Our previous studies also showed that adding lycopene to the diet of finishing pigs could significantly improve meat quality traits and regulate the conversion of muscle fiber types (Wen et al. 2022), but the regulatory effect of lycopene on skeletal muscle differentiation has not been reported yet. Additionally, estrogen receptor α (ERα or Esr1), one of the two canonical estrogen receptors, is the most abundantly expressed isoform in skeletal muscle and is regarded as an important signaling factor contributes to muscle health (Ribas et al. 2016; Z. Zhao et al. 2021). Recent studies also found that ERα showed a positive effect on promoting differentiation and myotube formation of myocytes (Huang et al. 2025). Overall, the activation of ERα signaling in skeletal muscle appears to exert a favorable effect (Sullivan et al. 2024). Interestingly, lycopene could induce the osteogenic differentiation process by activating the ERα/PI3K/AKT signaling pathway for protecting osteoporosis (Zhao et al. 2025). Nevertheless, whether lycopene could regulate myoblast differentiation through the ERα signal remains unknown.

In this study, we hypothesized that lycopene could promote myoblast differentiation and this function might be boosted by ERα activation. This research mainly investigates the interaction between lycopene and ERα, and aims to explore the potential roles of lycopene and ERα in myoblast differentiation.

## 2 Materials and Methods

### 2.1 Ethics Statement

Animal procedures were approved by the Animal Care Advisory Committee of Sichuan Agricultural University under permit No. YYS191220, and they supervised the whole experimental procedures. The experimental animal facility is authorized under license number CD‐SYXK‐2017‐015.

### 2.2 Animal Experiments

All mice (4 weeks old, male, BALB/c mice) were maintained under a stable temperature (22°C ± 2°C) and kept on a 12 h light/12 h dark cycle with free access to food and water. After acclimating for 3 days, all mice, purchased from GemPharmatech Co. Ltd. (Chengdu, China), were randomly divided into two groups. The control group (*n* = 18) was fed with the normal standard diet (met the nutrient recommendations for mice by AIN93G diet, Table 1). The Lycopene group (*n* = 18) fed the normal standard diet with lycopene (300 mg/kg, 300 mg of lycopene/kg diet) for 6 weeks. The dose of lycopene supplementation and the supplier of lycopene (purity: ≥ 98%, Xi'an Xiaocao Botanical Development Co. Ltd., Xi'an, China) was referenced to the previous studies (Wen et al. 2021). The normal standard diet obtained from Chengdu Dossy Experimental Animal Co. Ltd. (Chengdu, China). At the end of the experiment, the mice (except the mice used in the endurance assessment) were sacrificed and muscle samples were collected and measured.

**TABLE 1:** The formula of feeds for mice.

Ingredient	Diet (g/kg)	Nutrient levels	Proportion
Corn starch	397.50	Total energy, Kcal/g	3.96
Casein (≥ 85%)	200.00	Crude protein, %	17.50
Maltodextrin	132.00	Crude fat, %	7.00
Sucrose	100.00	Crude fiber, %	5.00
Soya‐bean oil	70.00	Carbohydrate, %	64.70
Cellulose	50.00		
Mixed mineral^a^	35.00		
Mixed vitamin^b^	10.00		
L‐cystine	3.00		
Choline tartrate	2.50		
Tert butyl hydroquinone	0.014		
Total	1000.00		

^a^ Contained following per kg of mineral mixture: Ca 5100 mg; P 3200 mg; K 3600 mg; Na 1300 mg; Fe 40 mg; Zn 35 mg; Mn 11 mg; Cu 6.0 mg; I 0.21 mg; Se 0.24 mg.

^b^ Contained following per kg of vitamin mixture: VA 4000 IU; VD3 1000 IU; VE 81.6 IU; VB1 thiamin hydrochloride 6.1 mg; VB2 6.7 mg; VB 2.1 mg; VB6 5.8 mg; VB12 29 μg; Niacin 30 mg; Pantothenic acid 16 mg; Biotin 0.2 mg; Choline chloride 1250 mg. Nutrient levels were calculated values.

### 2.3 Endurance Assessment

Five mice exhibiting average body weight were selected from each of the two groups. Endurance assessment was assessed using a motor‐driven open treadmill with a shock grid and the mice were acclimated to running on the treadmill (speed: 10 m/min; inclination: 15°) for 2 days. On the third day, exercise tolerance of the mice was tested. The mice were subjected to running under the acclimatization condition for 20 min. Every 2 min thereafter, the treadmill speed was increased by 2 m/min. Running was terminated when the mice contacted the shock grid for 3 s (Ahn et al. 2020).

### 2.4 Cell Culture and Treatment

C2C12 myoblast cells (ATCC, Manassas, VA, USA) were cultured at the stable condition (37°C, 5% CO_2_) and incubated in DMEM (Dulbecco modified Eagle medium) supplemented with 10% FBS, 100 U/mL penicillin, and 100 mg/L streptomycin (Gibco, Paisley, Scotland, UK). For verifying the effect of siRNA sequences, C2C12 cells (50% fusion) were transfected with si‐Esr1 sequences (GenePharma, Shanghai, China) using Lipo3000 (Invitrogen, USA). Myoblasts were harvested for RT‐qPCR analysis after 24 h. For the cell differentiation assay, approximately 80% fusion C2C12 cells were differentiated with differentiation medium (DM, DMEM supplemented with 2% HS) containing 0, 1, 2.5,and 5 μM lycopene for 3 days. For exploring the relationship between ERα and myogenic differentiation, cells were incubated with lycopene (2.5 μM) and ICI 182780 (1 μM, the estrogen receptor alpha inhibitor fulvestrant, purity: ≥ 98%, Topscience, Shanghai, China) for 3 days. For further mechanism studies, C2C12 cells were transfected with Lipo3000 and si‐Esr1 sequences and then induced to differentiate in the DM with 2.5 μM lycopene for 3 days. The lycopene used in in vitro assay was from Sigma (St. Louis, MO, USA) and its purity more than 98%.

### 2.5 Transcriptome Analysis and Identification of DEGs

The high‐quality RNA was extracted for subsequent analysis, and the RNA‐Seq and bioinformatics analysis were performed by Beijing Novogene Co. Ltd. (Beijing, China). The raw data had been deposited in NCBI SRA and the accession number was PRJNA1484312 (https://www.ncbi.nlm.nih.gov/sra/PRJNA1484312). Using the HISAT2 to align the filtered high‐quality reads (clean reads) to the reference genome. The expression level was calculated by using the FPKM of the reads. The PCA analysis (Principal component analysis) was conducted using log2 (FPKM+1) to perform a transformation and achieve rapid normalization of gene expression values. To identify the DEGs between the two groups, the DESeq2 software (version 1.20.0) was used to analyze the DEGs, the Benjamini & Hochberg method was adopted to adjust raw *p*‐value for controlling the FDR, and the criteria (|log2 (FoldChange)| ≥ 1.00 and adjusted *p*‐value < 0.05) was used to define the DEGs.

### 2.6 Functional Annotation and Enrichment Analysis

GO and KEGG analysis were conducted on the DEGs by clusterProfiler software (version 3.8.1). *p* value < 0.05 indicates that enrichment functions and pathways are significant.

### 2.7 H&E Staining

The H&E (hematoxylin–eosin) staining was performed by Wuhan sercicebio technology CO. LTD according to the SOP procedures for pathological examination in our unit. The muscles of mice were completely fixed in 4% paraformaldehyde, embedded in paraffin, sectioned at a suitable thickness, stained with H&E, and encapsulated. The images of stained sections were captured using Eclipse Ci‐L microscope, and the cross‐sectional area was quantified using Image‐Pro Plus 6.0 analysis software. Uniformly, using millimeters as the standard unit, count the number of muscle fibers in each tissue slice and measure the corresponding total cross‐sectional area of muscle fibers. Subsequently, calculate the average cross‐sectional area of individual muscle fibers by dividing the total cross‐sectional area by the total number of muscle fibers.

### 2.8 Enzyme Activity Assay

Mouse p‐AKT ELISA Kit and mouse p‐mTOR ELISA Kit were purchased from Shanghai enzyme‐linked Biotechnology Co. Ltd. (Shanghai, China) and used to measure p‐AKT and p‐mTOR concentrations in mice and in C2C12 myoblasts. The enzymatic activities of CK (creatine kinase, Cat. No. A032‐1‐1) were measured according to the manufacturer's instructions of Nanjing Jiancheng Bioengineering Institute.

### 2.9 Western Blotting

The protein samples in GAS tissues and C2C12 myotubes were extracted using the RIPA lysis buffer (produced by Beyotime, Shanghai, China). The BCA method was performed to assess the total protein concentrations. Total protein was fractionated on 8% SDS‐PAGE and transferred to PVDF membrane (Millipore, Eschborn, Germany) by electroblotting using wet Trans‐Blot system. Next, 5% bovine serum albumin was used to block the membranes (about 3 h at 37°C). Incubating the membranes with the primary antibodies at 4°C overnight: ERα (1:1000, Affinity, Cat# AF6058), β‐actin (1:1000, Cell Signaling, Cat. No. 4967). Then, the specific secondary antibodies were incubated the membranes at room temperature. BeyoECL Plus (produced by Beyotime, Shanghai, China) was used to visualize the purpose protein bands. The target bands were detected by ChemiDoc XRS Image System (Bio‐Rad, Hercules, CA, USA) and the target band densities were analyzed by Image J.

### 2.10 Immunofluorescence

According to the method from previous researches (Wen et al. 2020, 2021), the detailed method of immunofluorescence staining (IF) was as follows: C2C12 cells were treated with permeabilized buffer (Beyotime, China), blocked with blocking buffer (Beyotime, China), incubated with MHC primary antibody (1:50, MF‐20, DHSB) at 4°C for 24 h, and then incubated with the specific fluorescent‐labeled secondary antibodies at 37°C for 2.5 h. DAPI (4,6‐diamidino‐2‐phenylindole) was used to stain the nucleus. BA200 Digital microscope (Motic, Xiamen, China) was used to acquire all images. The fusion index was calculated based on the average number of cell nuclei in MyHC‐positive C2C12 cells.

### 2.11 RNA Extraction and RT‐qPCR

The E.Z.N.A. Total RNA Kit I reagent (Omega Bio‐Tek, USA) was used to extract high‐quality total RNAs. The HiScript III RT SuperMix for qPCR (+gDNA wiper) kit (Vazyme, Nanjing, China) was conducted a reverse transcription process, and then the ChamQ Universal SYBR qPCR Master Mix kit (Vazyme, Nanjing, China) was performed to relatively quantify mRNA. The quantitative real‐time PCR primer sequence is shown in Table S1. The PCR cycling conditions (40 cycles of 95°C for 15 s and 60°C for 60 s) were similar to the conditions in our previous study. The calculation formula for the relative gene expression level follows the 2^−△△Ct^ method, and GAPDH is used as the reference gene for normalization.

### 2.12 Statistical Analysis

All data are expressed as means ± the standard deviation (SD). SPSS 26.0 software was used for statistical analysis. Unpaired *t*‐test was used to analyze the significance of the results from the in vivo experiment. One‐way analysis of variance and Duncan's multiple‐range test were used to analyze the significance of results of in vitro experiment. The in vitro experiments in this study were conducted in three independent biological replicates. All data were tested for normality and homogeneity of variances (Shapiro–Wilk and Levene tests, respectively) before analysis. *p* value < 0.05 was regarded as statistically significant among groups.

## 3 Results

### 3.1 Effect of Lycopene on Skeletal Muscle Mass, Myofiber Size, and Muscle Function in Mice

**FIGURE 1:**
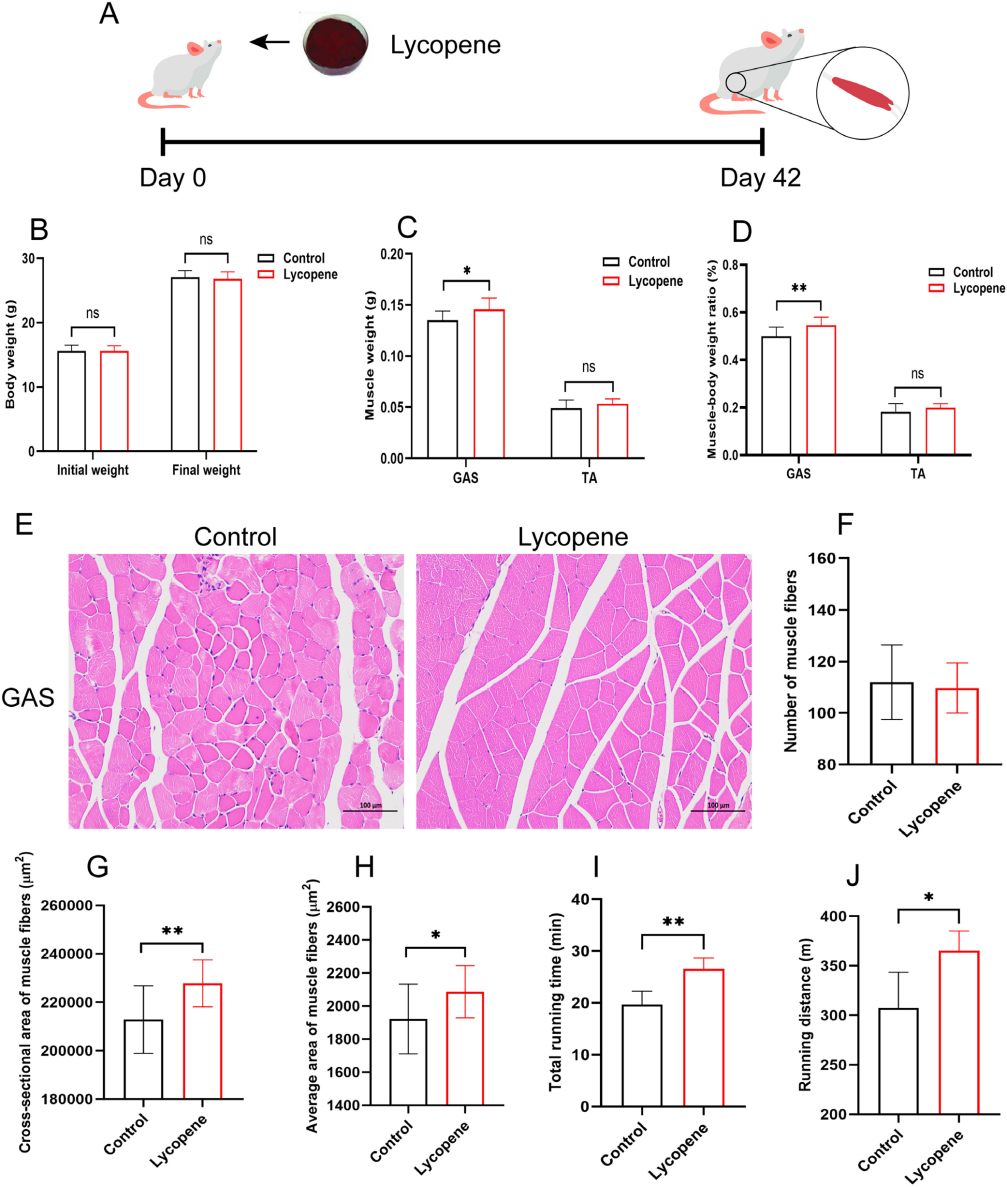
Effect of lycopene on skeletal muscle mass, myofiber size and muscle function in mice. Male BALB/c mice were fed the basal diet with or without 300 mg/kg lycopene for 6 weeks. (A) The experimental design of lycopene supplementation. (B) The body weight of initial weight and final weight of mice, *n* = 12. (C) Quantitative analysis of GAS and TA weight in mice and (D) The ratio of GAS and TA weight to body weight of mice, *n* = 12. (E) H&E staining of GAS, *n* = 4. Scar bar: 100 μm. Analysis results of H&E staining including (F) Number of muscle fibers, (G) Cross‐sectional of muscle fibers and (H) Average area of muscle fibers. (I) Total running time of mice from the experiment of endurance assessment, *n* = 5. (J) Running distance of mice from the experiment of endurance assessment, *n* = 5. The data were presented as the means ± SD. *p < 0.05 and **p < 0.01 as compared with control group.

The design of animal experiment in this research as shown in Figure 1A. Results indicated that addition of lycopene markedly increased the mass of GAS and significantly increased the proportion of GAS to whole body weight in mice compared to that in the control group, while there was no significance in the initial weight and final weight of mice (Figure 1B–D). Then, HE staining was used to detect the changes in the cross‐sectional area (CSA) of muscle fibers in GAS. We found that lycopene supplementation could significantly promote the CSA of muscle fibers in GAS (Figure 1E). Image‐Pro Plus 6.0 was used for analyzing the CSA of muscle fibers. As shown in Figure 1F–1H, the total CSA of muscle fibers and the average area of muscle fibers were larger in lycopene group than those in control group, while the number of muscle fibers showed no significant change. To evaluate the effect of lycopene on muscle performance, the Endurance assessment was performed. As shown in Figure 1I,J, lycopene supplementation significantly prolonged the total running time and increased running distance compared to that of the control group. These results suggested that lycopene supplementation significantly increased the skeletal muscle mass and enhanced muscle performance in mice.

### 3.2 Effect of Lycopene Supplementation on the Expression Levels of Muscle Protein Synthesis‐Related Factors and Myogenic Regulatory Factors in Mice

**FIGURE 2:**
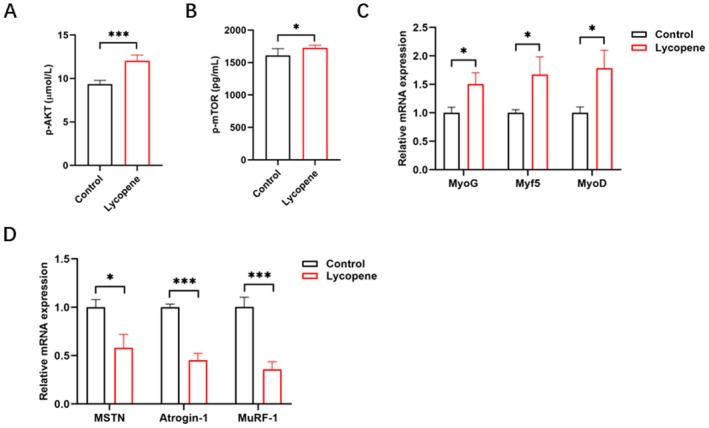
Effect of adding lycopene on the expression levels of muscle protein synthesis‐related factors and myogenic regulatory factors in mice. (A) p‐AKT concentration in mice (*n* = 6). (B) p‐mTOR concentration in mice (*n* = 6). (C) mRNA expressions of *MyoG*, *Myf5* and *MyoD* in GAS of mice (*n* = 4). (D) mRNA expressions of *MSTN*, *Atrogin‐1*, and *MuRF‐1* in GAS of mice (*n* = 4). The data were presented as the means ± SD. *p < 0.05 and ***p < 0.001 as compared with control group.

The mouse p‐AKT and p‐mTOR concentrations in GAS were evaluated using ELISA kits. The lycopene group exhibited a significant increase in the concentrations of p‐AKT and p‐mTOR (Figure 2A,B). The effect of lycopene supplementation on myogenic differentiation was assessed. The expressions of *MyoG*, *MyoD*, and *Myf5* were observably higher in the lycopene group than those in the control group (Figure 2C). Moreover, lycopene treatment significantly decreased the *MSTN* (*myostatin*), *MuRF‐1* (*muscle RING‐finger protein‐1*), and *Atrogin‐1* (*muscle atrophy F‐box*) expressions (Figure 2D). These results indicated that lycopene could significantly promote muscle differentiation and muscle protein synthesis in mice.

### 3.3 Quality Assessment of *RNA‐Seq* Data and Differentially Expressed Genes (DEGs) Analysis

**FIGURE 3:**
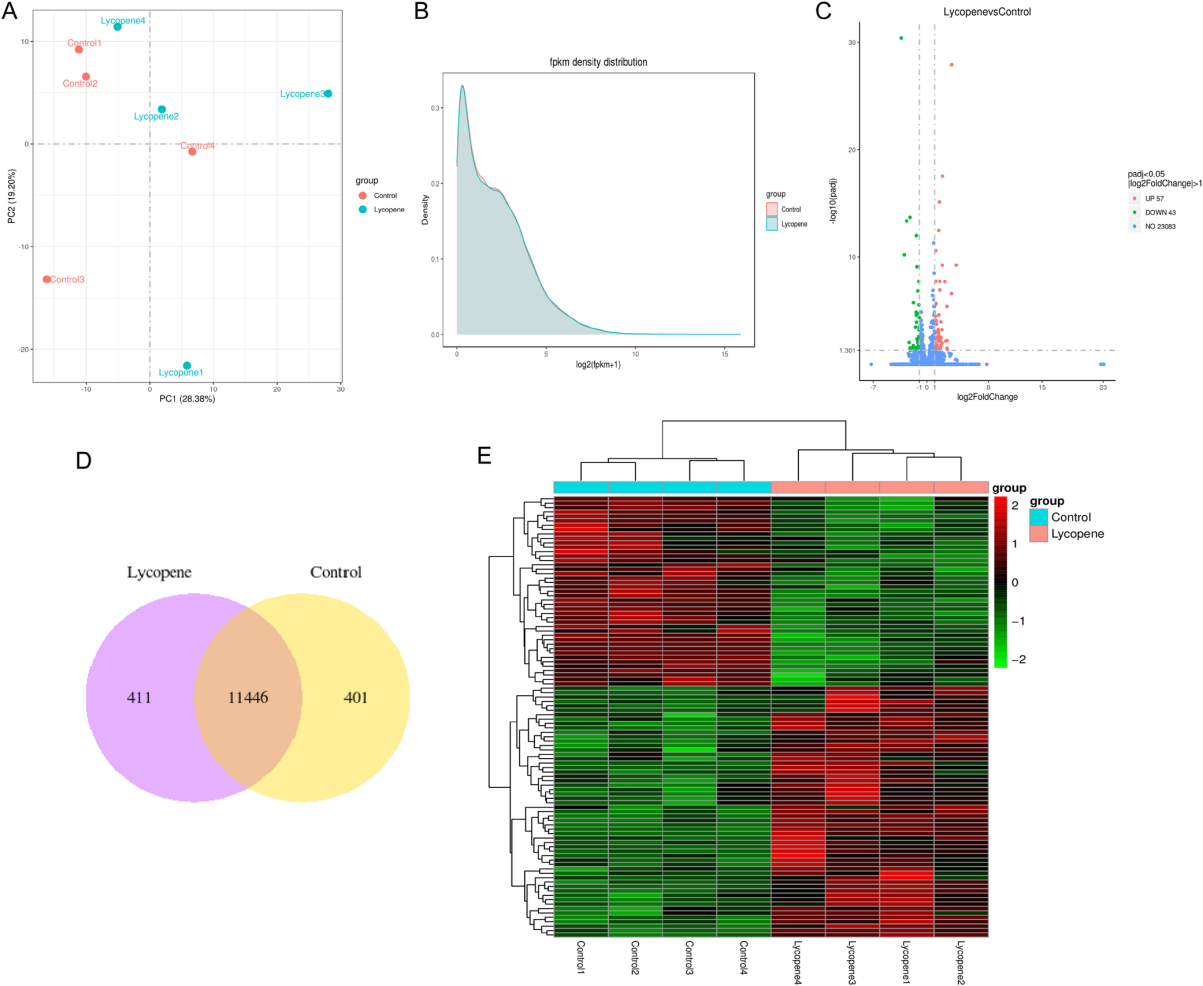
Quality assessment of RNA‐seq data and differentially expressed genes analysis (A) Principal component analysis (PCA) of RNA‐sequencing data (*n* = 4). (B) The expression distribution of all samples in control group and lycopene group (*n* = 4). (C) Volcano plot representing differentially expressed genes (DEGs, 100 genes), including 57 up‐regulated genes and 43 down‐regulated genes in GAS of mice between control group and lycopene group with |log2 (FoldChange)| ≥ 1.00 and adjusted *p*‐value < 0.05 (*n* = 4). (D) Venn diagrams showing the number of DEGs among control group and lycopene group (*n* = 4). (E) Heatmap of DEGs based among groups (*n* = 4). The horizontal axis represents sample names, and the vertical axis denotes normalized FPKM values of DEGs. Redder color indicates higher gene expressions, while greener color corresponds to lower expression levels.

To explore the potential mechanisms underlying lycopene supplementation‐induced skeletal muscle differentiation in mice, transcriptomic analysis was performed in the lycopene and control groups. Quality control of the sequencing data was conducted to ensure the accuracy of the following results. Results showed that the clean data of each sample ranged from 6.47 to 7.42 G, the Q30 percentage exceeded 95.29%, and the GC content varied between 47.74% and 50.22% (Table S2). 81.85%–87.29% of the reads from different samples were mapped to the reference genome after screening out low‐quality reads (Table S2). PCA (Principal component analysis) was used to analyze the transcriptomic data. Results found significant differences between the two groups, and the PC 1 (28.38%) × PC 2 (19.20%) indicated that samples of each group clustered together (Figure 3A). These results suggested that high‐quality RNA‐seq data from samples we prepared were obtained.

The FPKM (fragments per kilo‐base per million reads) was used to assess the expressions of all genes (Figure 3B). A total of 100 DEGs between lycopene group versus control group were identified, of which 57 genes were upregulated and 43 genes were downregulated (Figure 3C). Meanwhile, 441/401 unique DEGs of lycopene group/control group and 11,446 common genes were identified (Figure 3D). Analysis of DEGs clustering patterns indicated that the samples with the same group had approximately similar expression patterns (Figure 3E).

### 3.4 GO Enrichment and KEGG Pathway Analysis

**FIGURE 4:**
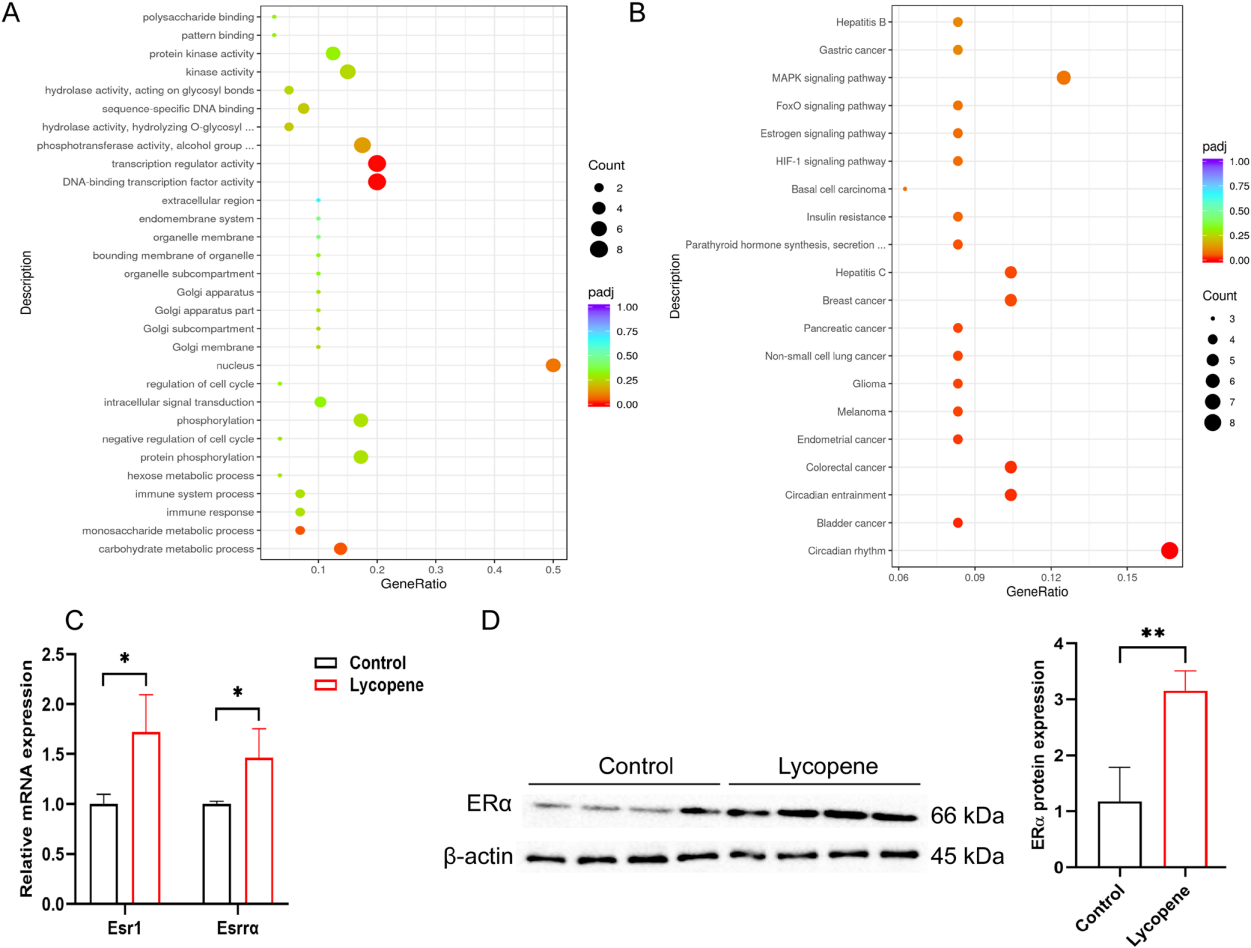
GO enrichment and KEGG pathway analysis. (A) GO (Gene Ontology) analysis of the functions of DEGs (*n* = 4). The vertical axis shows GO terms. The size of each dot indicates the number of genes annotated to the corresponding GO term, and the color gradient from red to purple illustrates the enrichment significance. (B) KEGG (Kyoto Encyclopedia of Genes and Genomes) enrichment analysis of the signaling pathways of DEGs (*n* = 4). The mRNA and protein expression levels of estrogen receptor α (ERα or Esr1) in GAS of mice were determined using RT‐qPCR and Western blotting. (C) mRNA expressions of *Esr1*, *Esrrα* in GAS of mice (*n* = 4). (D) Protein expressions of ERα in GAS of mice (*n* = 4). The data were presented as the means ± SD. *p < 0.05 and **p < 0.01 as compared with control group.

GO and KEGG analysis were used to functionally classify the DEGs for further elucidating the underlying mechanisms by which addition of lycopene modulated muscle differentiation. Among them, GO analysis found that most DEGs were enriched in the “molecular function” category, such as DNA‐binding transcription factor activity, transcription regulator activity and phosphotransferase activity (Figure 4A). The top enriched KEGG term was Estrogen signaling pathway, which is closely associated with muscle differentiation (Figure 4B). The KEGG analysis result was verified using the methods of real‐time quantitative PCR and Western blot, and we found that lycopene effectively upregulated *Esr1* and *Esrrα* mRNA levels and upregulated the protein level of ERα in GAS of mice (Figure 4C,D). These results suggested that lycopene supplementation could activate the ERα/ERRα axis in GAS of mice. It provides a novel insight to uncover the relationship between lycopene and muscle differentiation.

### 3.5 Effect of Lycopene on Differentiation of C2C12 Myoblast

**FIGURE 5:**
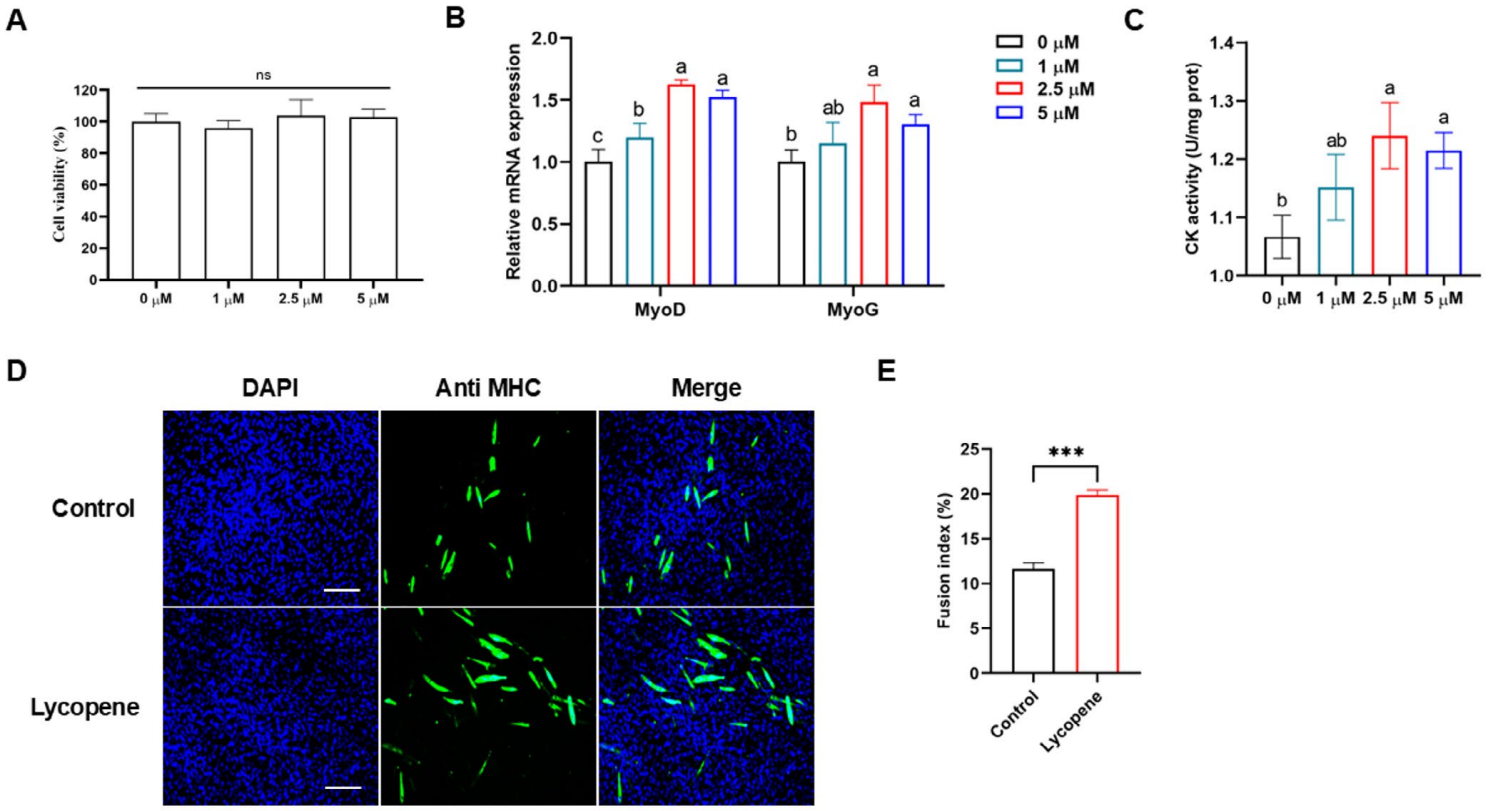
Effect of lycopene on differentiation of C2C12 myoblast. After C2C12 cells reached approximately 80% confluence, the myoblasts were induced to differentiate with differentiation medium containing 0, 1, 2.5, and 5 μM lycopene for 3 days. (A) Cell viability was measured by CCK‐8 (*n* = 6). (B) mRNA expressions of *MyoD* and *MyoG* in C2C12 cells (*n* = 3). (C) CK activity of C2C12 cells (*n* = 3). (D) Immunofluorescence staining of myosin heavy chain (MHC) on C2C12 cells. Scar bar: 100 μm. (E) Fusion index. The data were presented as the means ± SD. ***p < 0.001 as compared with control group. ^a‐c^Columns with variant alphabetical superscript mean significant differences (*p* < 0.05).

The potential role of lycopene in myogenic differentiation was investigated. As shown in Figure 5A, the cell viability of C2C12 myoblasts was measured using the Cell Counting Kit‐8 (CCK‐8), and results indicated that 0, 1, 2.5, and 5 μM lycopene were non‐toxic and not promote cell proliferation. *MyoD* and *MyoG* were significantly upregulated at the mRNA level in the lycopene group (Figure 5B). CK activity is regarded as a well‐established biomarker for C2C12 differentiation; therefore, we subsequently detected the effect of lycopene supplementation on CK activity. The results found that CK activity was significantly increased after lycopene treatment in differentiating C2C12 cells (Figure 5C). In addition, immunofluorescence staining of MHC indicated that lycopene supplementation obviously increased the MHC‐positive cell numbers and facilitated myocyte fusion (Figure 5D,E). These data revealed that lycopene promoted the differentiation of C2C12 myoblasts.

### 3.6 Effect of Lycopene on the Levels of Muscle Protein Synthesis‐Related Factors in C2C12 Myoblasts

**FIGURE 6:**
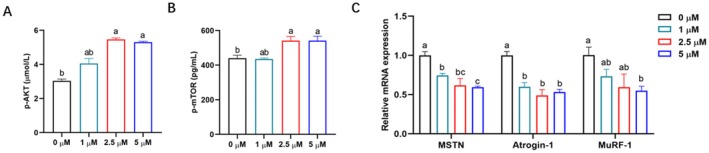
Effect of lycopene on the levels of muscle protein synthesis‐related factors in C2C12 myoblasts. (A) p‐AKT concentration in C2C12 myoblasts (*n* = 3). (B) p‐mTOR concentration in C2C12 myoblasts (*n* = 3). (C) mRNA expressions of *MSTN*, *Atrogin‐1* and *MuRF‐1* in C2C12 myoblasts (*n* = 3). The data was presented as the means ± SD. ^a‐c^Columns with variant alphabetical superscript mean significant differences (*p* < 0.05).

To assess the effect of lycopene on protein synthesis‐related factors, the concentrations of p‐AKT and p‐mTOR in C2C12 myoblasts were measured. Treatment with 2.5 and 5 μM lycopene upregulated the levels of p‐AKT and p‐mTOR in C2C12 myoblasts (Figure 6A,B). The mRNA levels of *MSTN*, *Atrogin‐1* and *MuRF‐1* were lower in the lycopene group than in the control group (Figure 6C). Taken together, 2.5 μM lycopene was selected for subsequent experiments.

### 3.7 Activation of ERα Promotes Myoblast Differentiation and Myotube Formation

**FIGURE 7:**
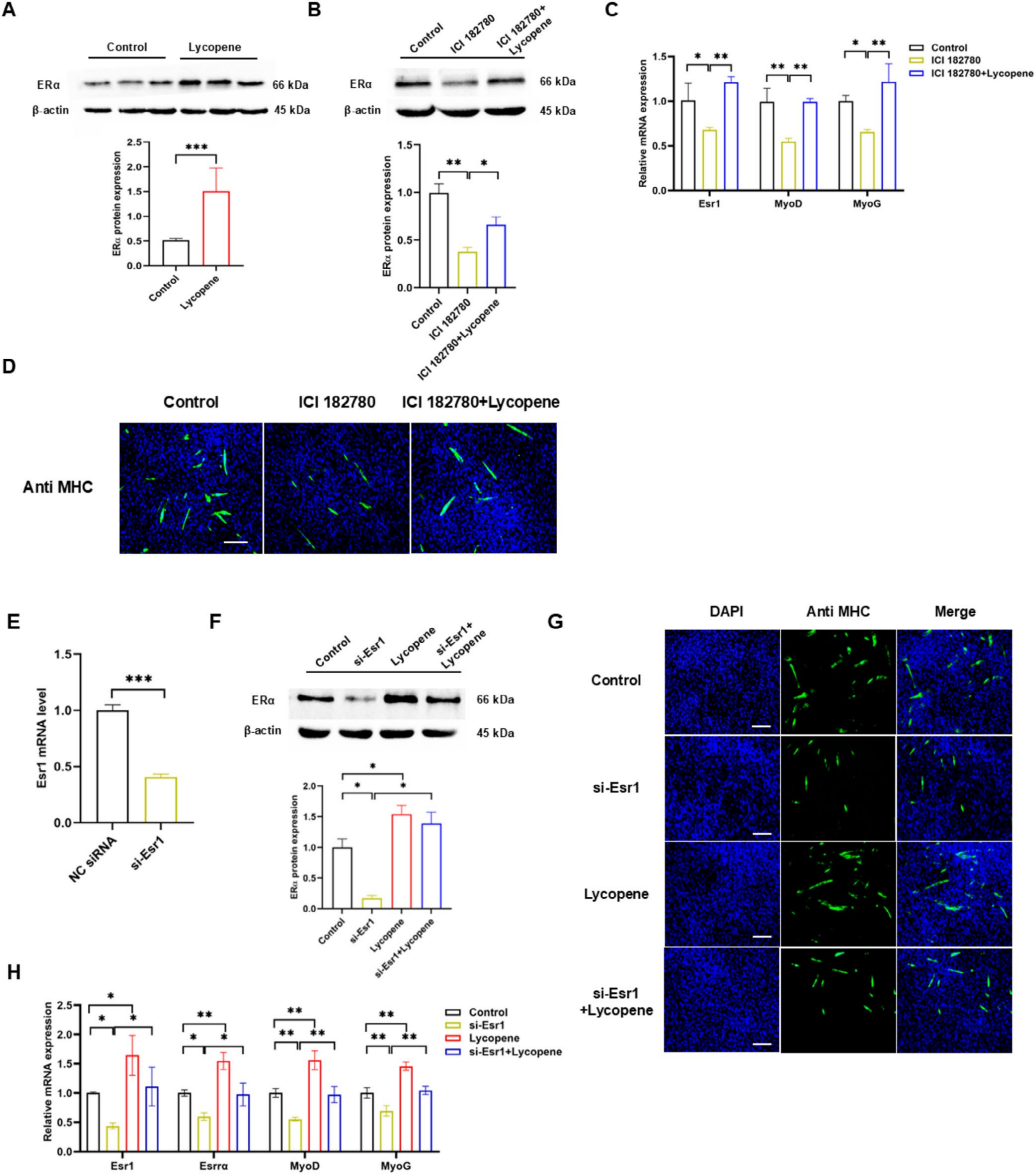
Activation of ERα promotes myoblast differentiation and myotube formation. (A, B) C2C12 cells (80% fusion) were treated with the differentiation medium (DM) with 2.5 μM lycopene or 1 μM ICI 182780 (the estrogen receptor alpha inhibitor fulvestrant) for 3 days. Western blotting measured the protein expressions of ERα after treatment with lycopene or ICI 182780 (*n* = 3). (C) Real‐time quantitative PCR measured *Esr1*, *MyoD*, and *MyoG* mRNA levels after treatment with lycopene or ICI 182780 (*n* = 3). (D) Immunofluorescence staining of anti‐MHC on C2C12 myotubes after treatment with lycopene or ICI 182780. (E) C2C12 cells (50% fusion) were transfected with Lipo3000 and si‐Esr1 sequences and then induced to differentiate in the differentiation medium (DM) with 2.5 μM lycopene for 3 days. mRNA expression of ERα in C2C12 cells after the transfection of si‐Esr1 sequence (*n* = 3). (F) Western blotting measured the protein expressions of ERα after transfection of si‐Esr1 and the supplementation with lycopene (*n* = 3). (G) Immunofluorescence staining of anti‐MHC on C2C12 myotubes after transfection of si‐Esr1 and the supplementation with lycopene. Scar bar: 100 μm. (H) mRNA expressions of *Esr1*, *Esrrα*, *MyoD*, and Myo*G* in C2C12 cells after transfecting with si‐Esr1 sequence and the administration of lycopene (*n* = 3). The data were presented as the means ± SD. *p < 0.05, **p < 0.01 and ***p < 0.001 as compared with control group.

This research mainly investigated the interaction between lycopene and ERα and explored whether lycopene could activate ERα to further promote myoblast differentiation. We found that lycopene supplementation effectively increased the protein level of ERα in C2C12 myotubes (Figure 7A). In order to confirm the important role of ERα in inducing myoblast differentiation, the estrogen receptor alpha inhibitor fulvestrant (1 μM ICI 182780) was administered during the induction of differentiation. The data found that fulvestrant downregulated the protein level of ERα compared to the control group; however, such decrease was attenuated through the treatment of lycopene (Figure 7B). Meanwhile, fulvestrant downregulated the mRNA expression levels of *Esr1*, *MyoD*, and *MyoG* and hindered myoblast differentiation, whereas lycopene partially counteracted fulvestrant‐induced differentiation inhibition (Figure 7C,D).

To investigate the further mechanism of lycopene‐promoted myogenic differentiation, C2C12 myotubes were transfected with siRNA specific for knockdown of ERα. The expression of ERα was sufficiently knocked down by siRNA in C2C12 myoblasts, while lycopene treatment significantly eliminated the inhibitory effect (Figure 7E,F). IF staining of MyHC showed that lycopene could promote myocyte fusion in si‐Esr1‐transfected C2C12 myoblasts (Figure 7G). As shown in Figure 7H, the inhibition of ERα signaling by si‐Esr1 significantly attenuated the promoting effects of supplementation lycopene on the gene expressions of *MyoD*, *MyoG*, and *Esrrα*. These data suggested that knockdown of ERα in myoblasts inhibited C2C12 differentiation and revealed that lycopene supplementation acted by activating ERα in myoblasts to promote myogenic differentiation and myocyte fusion.

## 4 Discussion

**FIGURE 8:**
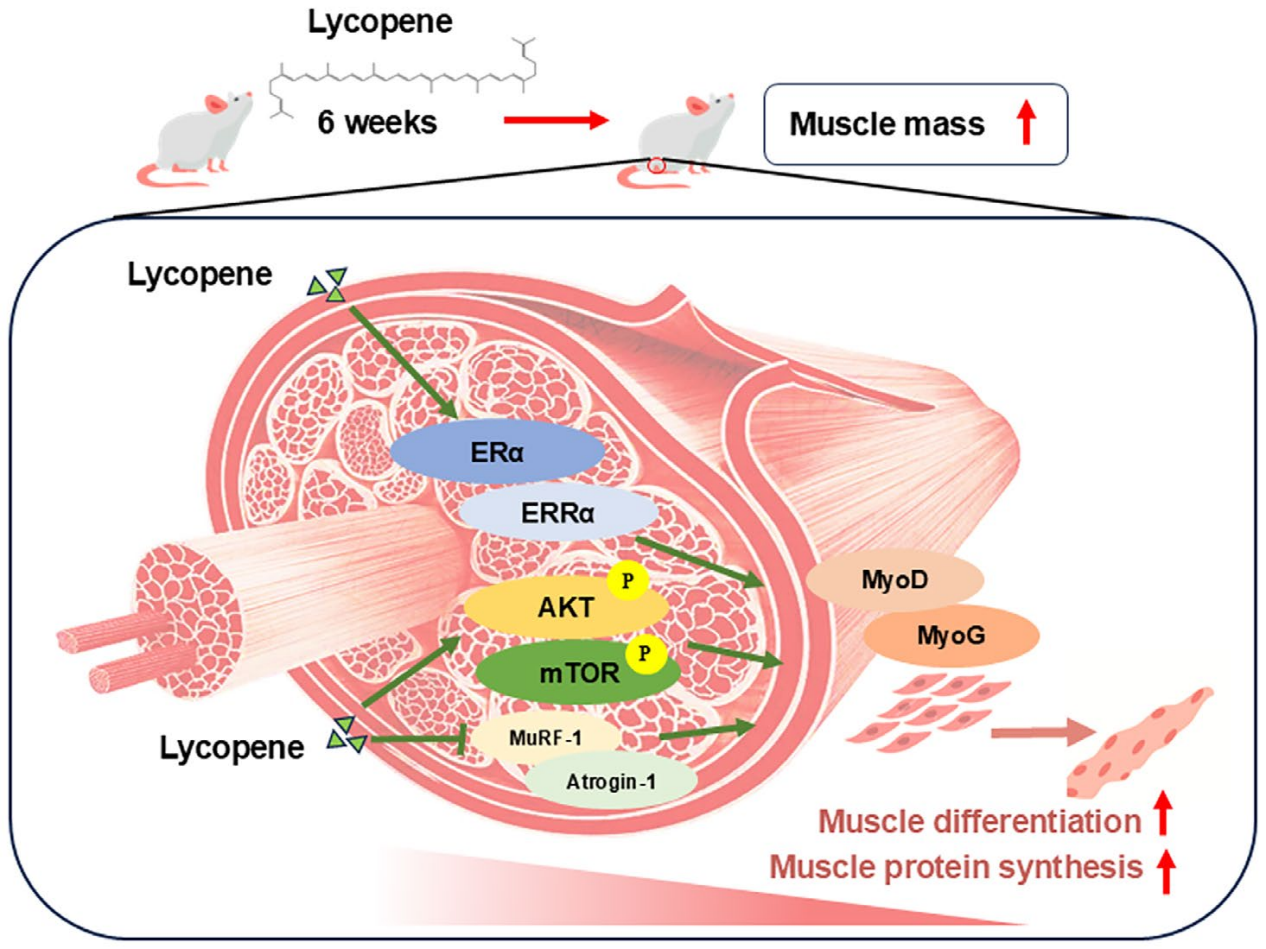
Schematic summary of the potential mechanisms of lycopene as nutritional factors in increasing muscle mass.

Skeletal muscle plays a critical role in locomotion and metabolic regulation, and maintaining adequate muscle mass is essential for healthy aging. The age‐ or disease‐related loss of muscle functional impairment can be mitigated through nutritional interventions, which may due to increased muscle mass (Kunkel et al. 2011; Liu, Sun, et al. 2021; Liu, Yang, et al. 2021; Montesano et al. 2013). This research has shown that lycopene, a natural antioxidant, promotes myoblast differentiation and muscle protein synthesis and increases muscle mass in in vivo experiments and in vitro assays. As shown in Figure 8, the schematic summary illustrated the potential mechanisms of lycopene as nutritional factors in increasing muscle mass. These findings in this study provide novel mechanistic insights into the potential of lycopene as a natural dietary supplement for supporting skeletal muscle homeostasis and promoting muscle health. Our previous studies had demonstrated that dietary supplementation with lycopene at 300 mg/kg (300 mg of lycopene/kg diet) significantly promoted the formation of slow‐twitch muscle fibers and improved muscle function in male BALB/c mice, whereas supplementation at 100 mg/kg yields no remarkable effects. Another research reported that male C57 BL/6 mice were fed with high‐fat diet mixed with 0.33% *w*/*w* lycopene for 8 weeks, and high‐fat diet‐induced dyslipidemia and oxidative stress were attenuated after lycopene supplementation. Additionally, to investigate the absorption efficiency of lycopene in human intestinal cells and in mice, male C57 BL/6 mice were fed ~6 g/day of a lycopene‐enriched diet (0.25 g of lycopene/kg diet) for 30 days. No evidence of toxicity of lycopene had been obtained from in vivo studies using laboratory animals. In summary, the in vivo experimental design of this study was as follows: the mice fed a lycopene (300 mg/kg)‐containing diet for a period of 6 weeks (Liu, Sun, et al. 2021; Liu, Yang, et al. 2021; Moussa et al. 2008; Wen et al. 2021). In this study, the average daily feed intake of the mice was approximately 4 g, resulting in a daily lycopene intake of approximately 1.2 mg. Notably, lycopene exhibits high biological safety and tolerance in vivo. Oral administration of lycopene at a daily dose of up to 100 mg causes no obvious adverse effects in healthy volunteers, and no toxic responses have been observed in laboratory animal models following lycopene treatment. Collectively, the low toxicity and high biocompatibility of lycopene provide flexible and feasible options for nutritional supplementation (Przybylska 2020). The in vivo experiments found that supplementation with lycopene led to significant increases in GAS mass, but not in TA mass. The GAS muscle is mainly composed of slow‐twitch fibers and is particularly important for anti‐fatigue ability (Lee et al. 2025). This muscle type‐specific effects could explain the observed increase in running time and the extension of running distance in the treadmill experiment. Moreover, lycopene not increased the final body weight of mice, suggested that the increase in muscle mass was not due to the increase in the overall weight of mice.

Muscle differentiation is integral component of the complex process of muscle growth and development (Chen et al. 2025; Zhang et al. 2024), and mainly controlled by MRFs (Jiang et al. 2023). Myf5 and MyoD promote the proliferation and differentiation of myogenic progenitor cells into myocytes, participating in the initial stage of muscle development; at the same time, MyoD is also crucial for maintaining the balance of protein synthesis and degradation, and ensuring the normal structure and function of the muscles (Kassar‐Duchossoy et al. 2004; Sun et al. 2008; Wang et al. 2024; Yamamoto et al. 2018). MyoG plays a crucial role in the process of myoblasts differentiating into myotubes and MHC is regarded as the terminal differentiation marker of myoblasts (Pette and Staron 2000; Zammit 2017). In this work, we confirmed that lycopene supplementation upregulated the mRNA levels of *MyoD*, *Myf5*, and *MyoG* in mice, and increased the mRNA levels of *MyoD* and *MyoG* in cells. Meanwhile, lycopene supplementation increased the MHC‐positive cell numbers, increased the activity of creatine kinase and promoted myotube formation in in vitro assays. These results indicated that lycopene supplementation promoted muscle differentiation in vivo and in vitro.

The differentiation of skeletal muscles occurs during the embryonic development stage, while the regeneration and repair of skeletal muscles occur throughout the entire life cycle. This process is particularly important during the aging process, as the loss of skeletal muscle mass caused by sarcopenia must be compensated for through myogenesis (Son et al. 2023). The results of this study showed that lycopene supplementation not only increased muscle mass but also promoted myogenic differentiation. This phenomenon indicated that supplementing with lycopene was beneficial for myogenesis, especially in cases of muscle atrophy caused by aging and sarcopenia. However, further research is still needed to clarify whether lycopene supplementation can prevent muscle diseases related to muscle atrophy.

In addition to promoting myoblast differentiation, we further uncovered that lycopene supplementation could modulate skeletal muscle growth. Growth of skeletal muscle is primarily governed by skeletal muscle hypertrophy—a process driven by a net increase in muscle protein synthesis relative to protein degradation. Conversely, muscle atrophy occurs when the rate of muscle protein breakdown exceeds the rate of protein synthesis (Liu et al. 2025; Sartori et al. 2009). It has been reported that the AKT/mTOR signaling pathway was positively correlated with the protein synthesis system of skeletal muscle (Kim et al. 2025). The phosphorylation of AKT could stimulate muscle protein synthesis through directly activating the mTOR signal factor (Stitt et al. 2004). The activated mTOR, as a key regulator of skeletal muscle mass, controls myogenesis through promoting the expression of myogenic differentiation markers (Ge and Chen 2012; Kim et al. 2025). The in vivo experiments revealed that lycopene enlarged the CSA in mice. Furthermore, lycopene supplementation not only increased the phosphorylation levels of AKT and mTOR but also enhanced the expressions of *MyoD* and *MyoG*, indicating that lycopene supplementation could facilitate protein synthesis to boost muscle growth. Moreover, the improvements in myoblast differentiation markers highlighted the potential of lycopene to facilitate muscle repair and growth. MSTN, a negative regulator for skeletal muscle growth, inhibits the hypertrophy of muscle fibers and limits the total amount of muscle tissue (Chen et al. 2021). Atrogin‐1 and MuRF‐1 are muscle atrophy regulatory factors and closely related to MSTN. When MSTN upregulates the expression levels of Atrogin‐1 and MuRF‐1 in myotubes, it reduces the levels of myogenic differentiation markers, thereby inhibiting muscle differentiation (Li et al. 2025; Singh et al. 2021). Interestingly, we further found that lycopene downregulated *MSTN*, *Atrogin‐1*, and *MuRF‐1*. Therefore, our study suggested that the inhibitory effect of lycopene on muscle protein degradation was beneficial for muscle growth.

Another novel finding of this study is that lycopene‐induced myogenic differentiation was strongly correlated with the estrogen signaling pathway, predicted by transcriptome sequencing analysis. Previous researches had reported that ERα could participate in diverse biological processes through the regulation of transcriptional activity of target genes and is widely expressed across various tissues and cell types (Shi et al. 2020). Estrogen‐related receptor α (ERRα or Esrrα) is downstream of ERα, and ERα could regulate the expression of ERRα, thereby influencing the expression of myogenic differentiation marker MyoD. Previous research had been reported that ERα‐mediated skeletal muscle regeneration via the ERα/ERRα/MyoD pathway and preventing muscle atrophy in *mdx* mice (Huang et al. 2025; Nguyen et al. 2025). Additionally, increasing evidence indicated that the ERα signal is regulated by nutrients (Yang et al. 2024; Zhao et al. 2025). Interestingly, in this research, the results confirmed that lycopene supplementation significantly increased the expression levels of ERα and its target gene in vivo and in vitro. These data illustrated that lycopene could activate ERα. Furthermore, treatment of C2C12 myoblasts with the ERα inhibitor ICI 182780 revealed that the pro‐myogenic effect of lycopene relied on ERα expression. Specific knockdown of ERα via ERα siRNA attenuated the promotional effects of lycopene on myoblast differentiation and ERα expression, and also abolished the upregulation of ERα downstream target genes *Esrrα* and *MyoD* induced by lycopene in in vitro assay. Collectively, these findings preliminarily demonstrated that lycopene activated ERα, and ERα‐activation facilitated myogenic differentiation and myotube formation. Nevertheless, ERα was not the only signaling pathway involved in lycopene‐induced myogenic differentiation. Moreover, this study only selected male mice as the experimental subjects, revealing the influence of lycopene on ERα and myogenic differentiation, but did not explore the relationship between lycopene and estrogen.

In conclusion, the results from murine models and C2C12 myoblast experiments firstly demonstrated that supplementation with lycopene could promote myogenic differentiation and muscle protein synthesis, contributing to increased muscle mass (Figure 8). This finding enriches the theoretical basis regarding lycopene as a nutritionally active compound regulating skeletal muscle development and myogenesis, and may possess potential for future research targeting human sarcopenia or muscle‐related disorders.

## Author Contributions

**Dajiang Ding:** resources, investigation. **Yexia Luo:** investigation, formal analysis. **Yong Zhang:** resources. **Daolin Mou:** conceptualization, methodology, software, data curation, supervision, funding acquisition, investigation, project administration, resources, writing – original draft. **Wanxue Wen:** methodology, investigation, validation, data curation, supervision, project administration, writing – original draft, resources, funding acquisition. **Jiaxin Du:** investigation, formal analysis. **Junning Pu:** investigation, resources, supervision.

## Funding

This work was supported by Sichuan Science and Technology Program (2024NSFSC1172, 2023NSFSC1140).

## Conflicts of Interest

The authors declare no conflicts of interest.

## Supporting information


**Table S1:** The quantitative real‐time (qRT‐PCR) primer sequence.
**Table S2:** Summary of quality preprocessing of RNA sequencing (RNA‐Seq) data.

## Data Availability

The data that support the findings of this study are available from the corresponding author upon reasonable request. The RNA‐seq data of this study have been deposited into the NCBI SRA under accession number PRJNA1484312.
