# Supplementary material for: Lycopene Increases Skeletal Muscle Mass by Promoting Myoblast Differentiation
Source: Food Sci Nutr. 2026 Aug 10;14(8):e72252. doi: 10.1002/fsn3.72252 (PMC13457663; doi:10.1002/fsn3.72252)
Supplement: Supplementary file 1 [file FSN3-14-e72252-s001.docx]

**Supplemental Table S1 The quantitative real-time (qRT-PCR) primer sequence**

| Origin | Gene | Primer | Sequence(5’to3’) |
| --- | --- | --- | --- |
|  | Esrrα | Forward | ACAGTGACAGTGGCACATGC |
|  |  | Reverse | AATCCAGATCCTCCAGCACA |
|  | Esr1 | Forward | TCCAGCAGTAACGAGAAAGGA |
|  |  | Reverse | AGCCAGAGGCATAGTCATTGC |
|  | MyoD | Forward | CCACTCCGGGACATAGACTTG |
|  |  | Reverse | AAAAGCGCAGGTCTGGTGAG |
|  | MyoG | Forward | GAGACATCCCCCTATTTCTACCA |
| Mouse |  | Reverse | GCTCAGTCCGCTCATAGCC |
|  | Myostatin | Forward | AGTGGATCTAAATGAGGGCAGT |
|  |  | Reverse | GTTTCCAGGCGCAGCTTAC |
|  | Myf5 | Forward | AAGGCTCCTGTATCCCCTCAC |
|  |  | Reverse | TGACCTTCTTCAGGCGTCTAC |
|  | MuRF-1 | Forward | CACTTTTCTACGTTGGTGCGAA |
|  |  | Reverse | CCCACCAAGGAAAGGAAGTAGG |
|  | Atrogin-1 | Forward | CTCTTTCTTGCCCGTCTTTTGG |
|  |  | Reverse | TTCCAGCCAAATGGAGAGAGAC |
|  | GAPDH | Forward | AGGGCATCTTGGGCTACAC |
|  |  | Reverse | TGGTCCAGGGTTTCTTACTCC |

**Supplemental Table S2 Summary of quality preprocessing of RNA sequencing (RNA-Seq) data**

| Sample | Raw reads | Clean bases | Q20 percentage (%) | Q30 percentage (%) | GC (%) | Mapped reads | Mapping rate  (%) |
| --- | --- | --- | --- | --- | --- | --- | --- |
| Control1 | 6.93G | 6.5G | 98.23 | 95.29 | 47.74 | 43319606 | 81.85% |
| Control2 | 6.96G | 6.47G | 99.12 | 97.34 | 48.74 | 43123596 | 82.95% |
| Control3 | 7.62G | 7.11G | 99.1 | 97.32 | 49.95 | 47415930 | 85.10% |
| Control4 | 7.99G | 7.42G | 99.1 | 97.3 | 49.73 | 49461762 | 85.23% |
| Lycopene1 | 7.71G | 7.17G | 99.13 | 97.36 | 50.22 | 47827202 | 87.17% |
| Lycopene2 | 7.51G | 7.14G | 99.13 | 97.39 | 49.32 | 47579628 | 84.11% |
| Lycopene3 | 7.55G | 6.9G | 99.08 | 97.27 | 50.09 | 46030188 | 87.29% |
| Lycopene4 | 7.09G | 6.51G | 99.1 | 97.33 | 49.43 | 43416018 | 84.12% |
